# Characterization of Lytic Bacteriophages Infecting Multidrug-Resistant Shiga Toxigenic Atypical *Escherichia coli* O177 Strains Isolated From Cattle Feces

**DOI:** 10.3389/fpubh.2019.00355

**Published:** 2019-11-26

**Authors:** Peter Kotsoana Montso, Victor Mlambo, Collins Njie Ateba

**Affiliations:** ^1^Bacteriophage Therapy and Phage Bio-Control Laboratory, Department of Microbiology, Faculty of Natural and Agricultural Sciences, North-West University, Mmabatho, South Africa; ^2^Food Security and Safety Niche Area, North-West University, Mmabatho, South Africa; ^3^Faculty of Agriculture and Natural Sciences, School of Agricultural Sciences, University of Mpumalanga, Mbombela, South Africa

**Keywords:** atypical enterophagenic *E. coli* O177, bacteriophages, bacteriophage therapy, biocontrol, biological properties, multi-drug resistance, shiga-toxigenic *E. coli*

## Abstract

The increasing incidence of antibiotic resistance and emergence of virulent bacterial pathogens, coupled with a lack of new effective antibiotics, has reignited interest in the use of lytic bacteriophage therapy. The aim of this study was to characterize lytic *Escherichia coli* O177-specific bacteriophages isolated from cattle feces to determine their potential application as biocontrol agents. A total of 31 lytic *E. coli* O177-specific bacteriophages were isolated. A large proportion (71%) of these phage isolates produced large plaques while 29% produced small plaques on 0.3% soft agar. Based on different plaque morphologies and clarity and size of plaques, eight phages were selected for further analyses. Spot test and efficiency of plating (EOP) analyses were performed to determine the host range for selected phages. Phage morphotype and growth were analyzed using transmission electron microscopy and the one-step growth curve method. Phages were also assessed for thermal and pH stability. The spot test revealed that all selected phages were capable of infecting different environmental *E. coli* strains. However, none of the phages infected American Type Culture Collection (ATCC) and environmental *Salmonella* strains. Furthermore, EOP analysis (range: 0.1–1.0) showed that phages were capable of infecting a wide range of *E. coli* isolates. Selected phage isolates had a similar morphotype (an icosahedral head and a contractile tail) and were classified under the order Caudovirales, *Myoviridae* family. The icosahedral heads ranged from 81.2 to 110.77 nm, while the contractile tails ranged from 115.55 to 132.57 nm in size. The phages were found to be still active after 60 min of incubation at 37 and 40°C. Incremental levels of pH induced a quadratic response on stability of all phages. The pH optima for all eight phages ranged between 7.6 and 8.0, while at pH 3.0 all phages were inactive. Phage latent period ranged between 15 and 25 min while burst size ranged from 91 to 522 virion particles [plaque-forming unit (PFU)] per infected cell. These results demonstrate that lytic *E. coli* O177-specific bacteriophages isolated from cattle feces are highly stable and have the capacity to infect different *E. coli* strains, traits that make them potential biocontrol agents.

## Introduction

Bacteriophages (phages) are self-replicating viruses, which are capable of infecting and lysing their specific host bacteria (1). They are ubiquitous organisms on Earth estimated to number at 10^30^-10^32^ (2). Phages are relatively safe, nontoxic, and harmless to animals, plants, and humans (3, 4). They are found in various environments related to their host such as in food, soil, sewage water, feces, and farm environments (2). Several bacterial species such as *Campylobacter, Escherichia coli, Listeria, Salmonella, Pseudomonas*, and *Vibrio* species are used as hosts to isolate their specific bacteriophages (5–7). Because of their host specificity and nontoxicity, lytic phages are considered to be an alternative solution to combat antimicrobial-resistant pathogens. Outbreaks of listeriosis and widespread occurrence of multidrug resistance in *E. coli, Salmonella*, and *Staphylococcus* species have been reported in South Africa (8–11). However, there has been no attempt to use bacteriophages to control antibiotic-resistant pathogens, in either hospital settings or food industry.

Antibiotic resistance in foodborne pathogens, particularly *E. coli* species, remains a public health concern. Antibiotic-resistant pathogens do not only increase economic and social costs but are also responsible for severe infections in humans (12). In 2014, foodborne infections caused an estimated 600 million illnesses and 420,000 deaths across the globe (13). In addition, 978 listeriosis cases were reported in South Africa from 2017 to 2018, resulting in 183 deaths (11). The leading pathogenic bacteria of concern are *E. coli* O157, *Campylobacter, Listeria*, and *Salmonella* species (14). Moreover, recent reports revealed that non-O157 strains, particularly O26, O45, O103, O111, O121, and O145, exhibit multidrug resistance and are among the leading causes of foodborne infection (15).

In view of the above, several interventions, such as physical, chemical, and biological methods, have been devised and implemented at all levels of the food chain to combat foodborne infection and the spread of antibiotic-resistant pathogens (16). However, these conventional methods have significant drawbacks such as corrosion of food processing plants, environmental pollution, change of food matrices, development of antibiotic resistance, and toxic effects of chemical residues (17). In addition, application of chemical agents coupled with a lack of effective enforcement regulations in food may hamper international trade and thus affect the economy of the exporting country (16, 18). Therefore, lack of new antibiotics and inefficacy of conventional strategies to combat multidrug-resistant bacterial pathogens necessitate the search for alternative control strategies such as the use of bacteriophages. Given their biological properties as explained above, lytic phages can be applied at all levels of the food chain, including preharvest application. Preharvest intervention has the advantage of preventing the transmission of foodborne pathogens from food-producing animals to human.

Considering the virulence and antibiotic resistance profiles of the *E. coli* O177 strain, coupled with the lack of new antibiotics and limitations of conventional strategies to mitigate antibiotic resistance, there is a need to expand the search for novel bacteriophages for biocontrol application. Therefore, the current study was designed to isolate and characterize lytic *E. coli* O177-specific bacteriophages as potential biocontrol agents. Stability and viability of the phages were determined under temperature and pH ranges that would be obtained in a live ruminant to assess their stability for preharvest use in these animals.

## Materials and Methods

### Bacterial Strain

Multidrug-resistant and virulent atypical enteropathogenic *E. coli* O177 strain was used to isolate *E. coli* O177-specific bacteriophages. The atypical enteropathogenic *E. coli* O177 isolates were obtained from cattle feces and confirmed through PCR analysis. The isolates were further screened for the presence of virulence and antimicrobial gene determinants. Prior to phage isolation, 40 *E. coli* O177 isolates stored at −80°C were resuscitated on MacConkey agar and incubated at 37°C for 24 h. A single colony from each sample was transferred into 15-ml nutrient broth in 50-ml falcon tubes. The samples were incubated in a shaker (160 rpm) at 37°C for 3 h until the growth reached an optical density (OD) of 0.4–0.5 (600 nm).

### Enrichment and Isolation Purification of *E. coli* O177-Specific Bacteriophages

*Escherichia coli* O177-specific bacteriophages were isolated from cattle feces using *E. coli* O177 environmental strain following the enrichment method (19, 20) with some modifications. Twenty fecal samples were collected from two commercial feedlots and two dairy farms. Samples were collected directly from the rectum using arm-length rectal gloves, placed in a cooler box containing ice packs, and transported to the laboratory. Five grams of each fecal sample was dissolved in 20 ml of lambda diluent and vortexed to obtain a homogeneous mixture. The mixture was centrifuged at 10,000 × *g* for 10 min using Hi Centrifuge SR (model: Z300, Germany) to sediment fecal matter and other impurities. An aliquot of 10 ml from the supernatant was extracted and filter-sterilized using a 0.22-μm pore-size syringe filter (GVS Filter Technology, USA) to obtain crude phage filtrates. For enrichment, 5 ml of each filtrate was added to 100 μl of exponential-phase (OD_600_ = 0.4–0.5) culture of each of the 40 *E. coli* O177 host strains in 10 ml of double-strength tryptic soya broth (TSB) supplemented with 2 mM of calcium chloride (CaCl_2_). The samples were incubated in a shaking incubator (80 rpm) at 37°C for 24 h. After incubation, the samples were centrifuged at 10,000 × *g* for 10 min using Hi Centrifuge SR (Model: Z300, Germany) to remove bacterial cells and sample debris. The supernatant was filter-sterilized with a 0.22-μm pore-size Acrodisc syringe filter (GVS Filter Technology, USA) to obtain crude phage filtrates.

Subsequently, a spot test was performed to determine the presence of phages as previously described (19). Briefly, 100 μl of exponential-phase (OD_600_ = 0.4–0.5) culture of the bacterial host was mixed with 3 ml of soft agar (0.3% w/v agar) held at 50°C, then poured onto modified nutrient agar (MNA) plates so as to create a bacterial lawn, and allowed to solidify for 15 min. Ten microliters of each crude phage filtrate was spotted on bacterial lawn, and the plates were incubated at 37°C for 24 h. After incubation, the plates were observed for the presence of clear zones or plaques at inoculated points. Plaques were picked using a sterile pipette tip and suspended in 1 ml of lambda diluent [10 mM of Tris Cl (pH 7.5), 8 mM of MgSO_4_·7H_2_O] in 2-μl Eppendorf tubes. The tubes were left at room temperature to allow phages to diffuse into the solution. The tubes were then centrifuged at 11,000 × *g* for 10 min, and the supernatant was filter-sterilized with a 0.22-μm pore-size Acrodisc syringe filter (GVS Filter Technology, Germany).

### Bacteriophage Purification and Propagation

Bacteriophages were purified from single plaque isolates using the soft agar overlay method (21, 22). Plaque assay was performed, and the plates were incubated at 37°C for 24 h. After incubation, single plaques from each plate were picked based on their sizes and clarity using a sterile pipette tip and were resuspended in 1 ml of lambda diluent in 2-μl Eppendorf tubes. The tubes were left at 4°C for 24 h to allow phage to diffuse into the buffer. The tubes were then centrifuged at 10,000 × *g* for 10 min, and the supernatant was filter-sterilized using a 0.22-μm pore-size Acrodisc syringe filter (GVS Filter Technology, Germany). The purification process was repeated three consecutive times until homogeneous plaques were obtained for each phage isolate. Purified phages were propagated using *E. coli* O177 host bacteria. One hundred microliters of pure phage stocks was mixed with 100 μl of exponential-phase (OD_600_ = 0.4–0.5) culture of corresponding host(s) in a 50-ml falcon tube containing sterile 10-ml double-strength TSB supplemented with 2 mM of CaCl_2_. The mixture was incubated in a shaking incubator (150 rpm) at 37°C for 24 h. After incubation, the samples were centrifuged at 8,000 × *g* for 10 min at 4°C, and the supernatant was filter-sterilized using a 0.22-μm pore-size Acrodisc syringe filter (GVS Filter Technology, Germany). Ten-fold serial dilutions were prepared, phage titers were determined using plaque assay, and the titers were expressed as PFU per milliliter. The stock phages were stored at 4°C for further analysis.

### Characterization of Selected *E. coli* O177-Specific Bacteriophages

#### Host Range and Cross Infectivity of the Phage Isolates

The host range of eight selected phage isolates was evaluated against 50 bacterial hosts [13 *E. coli* O177, 12 *E. coli* O157, 12 *E. coli* O26, and 10 *Salmonella* species (environmental strains), 1 *Pseudomonas aeruginosa* (ATCC 27853), 1 *Salmonella enterica* (ATCC 12325), and 1 *Salmonella typhimurium* (ATCC 14028)], and all environmental species were isolated from cattle feces. Phage isolates were selected based on different plaque morphologies and clarity of the plaques and sizes. The spot test technique was performed to determine lytic spectrum activity of each phage isolates as previously described (21). The bacterial lawns of all the selected bacterial hosts were prepared on MNA plates. Ten microliters of phage stock (10^7^–10^9^ PFU/ml) was spotted on bacterial lawn and allowed to air-dry under laminar airflow for 10 min. The plates were incubated at 37°C for 24 h. After incubation, the plates were observed for the presence of plaques at the point of application, and the phage lytic profiles were classified into three categories according to their clarity: clear, turbid, and no lysis (23). The test was performed in triplicates for each phage isolate.

#### Efficiency of Plating of Phages

Efficiency of plating (EOP) was performed to determine lytic efficiency of the phage in comparison with their suitable host bacteria as previously described (24), with modification. Fifteen bacterial strains (five *E. coli* O177, five *E. coli* O26, and five *E. coli* O157) were selected based on their sensitivity against the phages. Ten-fold serial dilutions of phages were prepared to obtain single plaques. An aliquot of 100 μl of each phage (1 × 10^4^ PFU/ml) was mixed with 100 μl of exponential-phase (OD_600_ = 0.4–0.5) culture of each bacterium in 50-ml sterile falcon tubes and left for 10 min at room temperature to allow the phage to attach to the host. Then, 3 ml of soft agar (0.3% w/v) was added to the tube, and the mixture was poured onto MNA plates. Three independent assays were performed for each phage isolate. After solidifying, the plates were incubated at 37°C for 24 h. After incubation, the number of plaques per plate was counted. The EOP was calculated as the ratio between the average number of plaques on target host bacteria (PFUs) and average number of plaques of reference host bacteria (PFUs). The EOP was classified as high (EOP ≥ 0.5), moderate (EOP > 0.01 < 0.5), and low (EOP ≤ 0.1) based on the reproducible infection on the targeted bacteria (25). The following formula was used to calculate EOP values:

$$\begin{array}{l} \text{Relative EOP}=\frac{\text{average number of plaques on targeted host bacteria }\left(\text{PFUs}\right)}{\text{average number of plaques on reference host bacteria }\left(\text{PFUs}\right)} \end{array}$$

### Transmission Electron Microscopy Analysis

Eight phage isolates were subjected to transmission electron microscopy (TEM), and phage morphotype was determined using negative staining techniques as previously described (26), with some modifications. Briefly, phages were propagated to obtain high titer (10^8^–10^11^ PFU/ml). Ten milliliters of each phage (10^8^–10^11^ PFU/ml) was concentrated in 50-ml falcon tubes by adding 10% (w/v) PEG, and the mixture was incubated at 4°C overnight to allow precipitation of the phage particles. After incubation, phage particles were sedimented by centrifugation at 11,000 × *g* for 10 min at 4°C. The supernatant was discarded, and the pellet was washed three times with 0.1 M ammonium acetate (pH 7.0). The pellet was resuspended in 200 μl of ammonium acetate. Ten microliters of concentrated phage solution was deposited on 200-mesh copper grids with carbon-coated formvar films. The phage particles were allowed to adsorb for 2 min, and excess liquid was drained off with a sterile filter paper. The grid was allowed to air-dry. A drop of 1% (w/v) ammonium molybdate (aqueous, pH 6.5) was added to negatively stain the phage particles and allowed to air-dry for 10–15 min. The grid containing the specimen (phage particles) was then loaded into a transmission electron microscope (model: FEI Tecnai; TEM, Czech Republic) and operated at 120 kV to scan and view phage images with a magnification range of 20,000–100,000. Micrographs were taken with a Gatan bottom-mounted camera using Digital Micrograph software at 80 kV and a magnification range of 20,000–250,000. The images were taken, and morphology characteristics were used to classify phage isolates as previously described (27).

### Effect of Different Temperatures and pH on the Stability and Viability of Phages

Phage stability and viability were evaluated across different temperatures (37 and 40°C) over a 60-min period in a temperature-controlled incubator. The concentration of the host bacteria and phage titers was standardized before starting the experiment. One hundred microliters of exponential-phase culture [10^5^ colony-forming unit (CFU)/ml] and 100 μl of phages (10^5^ PFU/ml) was added to 10 ml of double-strength TSB supplemented with 2 mM of CaCl_2_. The tubes were incubated in a preset shaking incubator at 37 and 40°C for 60 min, and samples were taken at 10, 30, and 60 min of incubation and assessed for viability and concentration using double-layer agar (22). Plaque assay was performed in triplicates for each sample, and the results were expressed as PFU per milliliter. For pH, phages were exposed to different pH levels (3.0, 4.5, 6.3, 7.0, 8.5, and 10.0) in a 48-h incubation period. Ten milliliters of sterile double-strength TSB (amended with 2 mM of CaCl_2_) was distributed into 50-ml falcon tubes to prepare different pH solutions. The pH was adjusted using hydrochloric acid (HCL, 6M) or sodium hydroxide (NaOH, 6M). One hundred microliters of each bacterial host (10^5^ CFU/ml) and their corresponding phage (10^5^ PFU/ml) isolates were added to 10 ml. The tubes were incubated in a preset shaking incubator (80 rpm) at 37°C for 48 h. Samples were taken at 24 and 48 h of incubation, and the phage titer for each sample was determined using the standard plaque assay. Plaque assay was performed in triplicates for each sample, and the results were expressed as PFU per milliliter.

### One-Step Growth Curve

A one-step growth curve experiment was performed to determine the latent period and burst size of the selected phages as previously described (21), with some modifications. Briefly, 5 ml of exponential-phase culture of each host was centrifuged at 8,000 × *g* for 5 min at 4°C. The pallet was resuspended in 10 ml of double-strength TSB supplemented with 2 mM of CaCl_2_ to obtain an OD of 0.4–0.5 (600 nm). The bacterial concentration was adjusted using sterile double-strength TSB to obtain 1 × 10^8^ CFU/ml. Each phage (10^8^ PFU/ml) was added to its respective host bacterial suspension to achieve multiplicity of infection (MOI) 1.0. The mixture was left at room temperature for 10 min to allow phages to adsorb to the host bacteria. After 10 min, 1.5 μl of the mixture was transferred into 2-μl Eppendorf tubes and centrifuged at 11,000 × *g* for 10 min to remove unadsorbed phage particles. The pellet was resuspended in 100 μl of TSB supplemented with 10 mM of magnesium sulfate (mTSB) and transferred into 9.9 ml of prewarmed mTSB. The samples were incubated in a shaking incubator (160 rpm) at 37°C for 1 h. Two hundred microliters was drawn from each sample at 5-min intervals for 60 min. Plaque assay was performed in triplicates for each samples to determine phage titer. The data generated were used to determine the latent period, burst time, and phage relative burst size per infected cell. The burst size was calculated as the ratio of the final count of released phage progeny to the initial count of infected bacterial host cell during the latent period using the following formula as previously described (28):

$$\begin{array}{l} \text{Relative burst size}=\frac{\text{final titer }\left(\text{PFU}\right)-\text{initial titer }\left(\text{PFUs}\right)}{\text{initial titer }\left(\text{PFUs}\right)\;\left(\text{PFUs}\right)} \end{array}$$

The relative burst size at different time points was plotted against time to determine the latent period and burst size of each phage isolate.

### Statistical Analysis

The viability and stability of phages were tested at different temperatures and pH levels. The data were converted to log_10_ PFU per milliliter and analyzed using SAS (2010). The effect of temperature, time, and phage type on viability and stability of phages was analyzed using the general linear model (GLM) procedure of SAS (2010) for a 2 (temperature) × 3 (time) × 8 (phages) factorial treatment arrangement according to the following model:

$$\begin{array}{l} Y_{ijkl}\;=\;\mu \;+\;T_{i}\;+\;S_{j}+V_{k}+{\left(T\times S\right)}_{ij}+{\left(T\times V\right)}_{ik}\;\\ \;+\;{\left(S\times V\right)}_{jk}\;+\;{\left(T\times S\times V\right)}_{ijk}\;+\;E_{ijkl} \end{array}$$

where *Y*_*ijkl*_ is the observation of the dependent variable *ijkl*; μ is the fixed effect of population mean for the variable; *T*_*i*_ is the effect of temperature; *S*_*j*_ is the effect of time; *V*_*k*_ is the effect of phages; (*T* × *S*)_*ij*_ is the effect of interaction between temperature at level *i* and time at level *j*; (*T* × *V*)_*ik*_ is the effect of interaction between temperature at level *i* and phage at level *k*; (*S* × *V*)_*jk*_ is the effect of interaction between time at level *j* and phage at level *k*; (*T* × *S* × *V*)_*ijk*_ is the effect of interaction between temperature at level *i*, time at level *j*, and phage at level *k*; and *E*_*ijkl*_ is the random error associated with observation *ijkl*.

Phage viability and stability data in response to incremental levels of pH were evaluated for linear and quadratic effects using polynomial contrasts. Response surface regression analysis (Proc RSREG; SAS 2010) was applied to describe the responses to pH according to the following quadratic model: *y* = *a* + *bx* + *cx*^2^, where *y* is the response variables, *b* and *c* are the coefficients of the quadratic equation, *a* is the intercept, *x* is the pH level, and –*b*/2*c* is the *x* value for maximum response. For all statistical tests, significance was declared at *p* ≤ 0.05.

## Results

### Isolation, Purification, and Propagation of Bacteriophages

**Figure 1:**
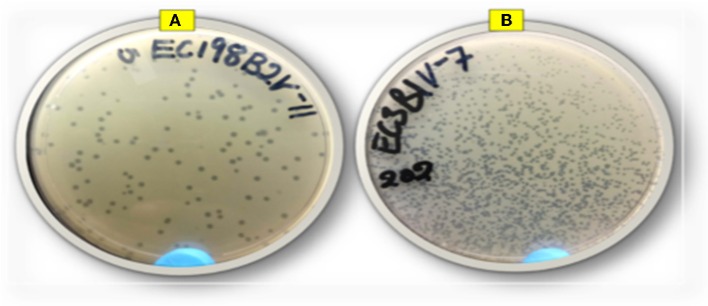
Representative image of phage isolates depicting different plaque morphologies: **(A)** EC198B2PV (large plaques) and **(B)** EC3B1PV (small plaques).

Thirty-one lytic *E. coli* O177-specific bacteriophages were isolated from cattle feces. Phages were able to infect 15% of the *E. coli* O177 isolates used for isolation. Phage isolates were designated as ECPV, according to the genus of the host bacteria, followed by the notation of phage virus and a numeric number as identity. Phage isolates revealed different plaque morphologies in terms of sizes, ranging from small to large (1–2 mm, respectively) plaques (Figure 1). A large proportion (71%) of the phage isolates revealed large plaques, while a small proportion (29%) showed small plaques on their preferred hosts. All the phages revealed clear (complete lysis) plaques, and no turbid plaques were observed. Phage titer after propagation ranged from 6.2 × 10^5^ to 3.1 × 10^13^ PFU/ml. Phage EC3A2PV had the lowest titer, while phage EC198B1PV had the highest titer compared to other phage isolates.

### Host Range of Phages and EOP Analysis Against Different *E. coli* Strains

**Figure 2:**
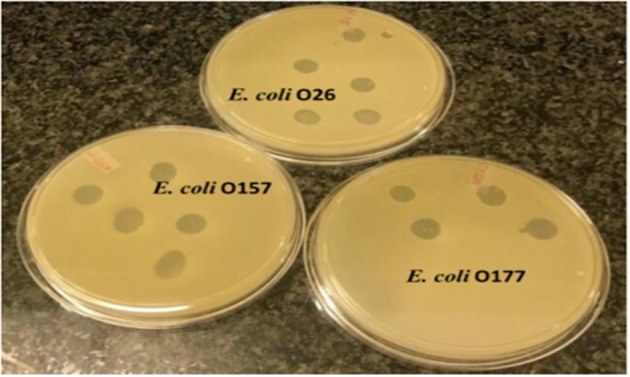
Representative image depicting spot test results of phages on different *Escherichia coli* strains.

A spot test was performed to determine the host range of eight selected lytic phage isolates against 50 bacterial hosts comprising different bacterial species. The results indicated that the phages were capable of infecting *E. coli* species (*E. coli* O177, *E. coli* 0157, and *E. coli* O26 environmental strains) tested (Figure 2). All phage isolates produced clear plaques against all *E*. *coli* O177 and 83–100% of *E. coli* O26 strains (Table 1). Three phages (EC10C3PV, EC11B2PV, and EC12A1PV) were able to infect *E. coli* O157 (75–83%; Table 2). None of the phages could infect ATCC strains and environmental *Salmonella* species. The EOP analysis was performed on 15 (five *E. coli* O177, five *E. coli* O26, and five *E. coli* O157) isolates that were susceptible to phages on the spot test. Although spot test results revealed clear plaques on *E. coli* O177, EOP results exhibited various lytic patterns of the phages. Even though EOP analysis revealed high (EOP ≥ 0.5) productive infection on *E. coli* O177, moderate infections were observed (Table 2). Four phages revealed high EOP values (0.5–0.8) on *E. coli* O177 isolates. On the other hand, EOP analysis exhibited moderate and low productive infections on *E. coli* O26 and *E. coli* O157 isolates (EOP values range from 0.0 to 0.4 and 0.0 to 0.3, respectively).

**Table 1:** Host range infection of the phages.

**Host bacteria**	**No**.	**Phage host range (%)**							
		**EC366VPV**	**EC11B2PV**	**EC10C2PV**	**EC12A1PV**	**EC3A1PV**	**EC118BPV**	**EC366BPV**	**EC10C3PV**
*Pseudomonas aeruginosa*^a^	1	1 (0%)	1 (0%)	1 (0%)	1 (0%)	1 (0%)	1 (0%)	1 (0%)	1 (0%)
*Salmonella enterica*^b^	1	1 (0%)	1 (0%)	1 (0%)	1 (0%)	1 (0%)	1 (0%)	1 (0%)	1 (0%)
*Salmonella typhimurium*^c^	1	1 (0%)	1 (0%)	1 (0%)	1 (0%)	1 (0%)	1 (0%)	1 (0%)	1 (0%)
*Escherichia coli* O177^d^	13	13 (100%)	13 (100%)	13 (100%)	13 (100%)	13 (100%)	13 (100%)	13 (100%)	13 (100%)
*E. coli* O26^d^	12	12 (100%)	10 (83%)	11 (92%)	11 (92%)	10 (83%)	11 (92%)	11 (92%)	11 (92%)
*E. coli* O157^d^	12	12 (0%)	9 (75%)	12 (0%)	10 (83%)	12 (0%)	12 (0%)	12 (0%)	10 (83%)
*Salmonella* species^d^	10	10 (0%)	10 (0%)	10 (0%)	10 (0%)	10 (0%)	10 (0%)	10 (0%)	10 (0%)

a, b, c, d *ATCC 27853, ATCC 12325, ATCC 14028, and environmental strains, respectively*.

**Table 2:** Efficacy of plating of phages against different *Escherichia coli* serotypes.

**Bacterial strain**	**Bacteria ID**	**EOP ratio of phage isolates**							
		**EC10C2PV**	**EC10C3PV**	**EC118BPV**	**EC11B2PV**	**EC12A1PV**	**EC366BPV**	**EC366VPV**	**EC3A1PV**
*E. coli* O177	CF-D-D202	0.7	0.8	0.6	0.5	0.7	0.8	0.8	1.0^a^
	CF-A27	0.5	0.6	0.7	0.7	1.0^a^	0.8	0.7	0.6
	CF-H361	1.0^a^	1.0^a^	0.5	0.6	0.8	0.8	0.6	0.7
	CF-A28	0.4	0.7	0.5	1.0^a^	0.5	0.5	1.0^a^	0.8
	CF-D-D246	0.7	0.9	1.0^a^	0.8	0.8	1.0	0.8	0.7
*E. coli* O26	2A	0.3	0.2	0.4	0.1	0.0	0.4	0.2	0.0
	4C	0.3	0.2	0.2	0.1	0.1	0.1	0.3	0.1
	17E	0.2	0.2	0.1	0.1	0.3	0.3	0.2	0.3
	21F	0.1	0.1	0.3	0.3	0.2	0.2	0.1	0.1
	25H	0.3	0.3	0.1	0.3	0.1	0.3	0.2	0.1
*E. coli* O157	1A	0.1	0.1	0.2	0.1	0.0	0.1	0.3	0.3
	3B	0.2	0.2	0.1	0.1	0.0	0.1	0.1	0.1
	5D	0.3	0.1	0.1	0.2	0.1	0.1	0.1	0.2
	7F	0.1	0.1	0.1	0.1	0.1	0.2	0.1	0.1
	8G	0.1	0.3	0.1	0.3	0.1	0.2	0.1	0.1

*ID, identity*.

a *Reference host*.

### Morphological Characterization of Phages Based on TEM

**Figure 3:**
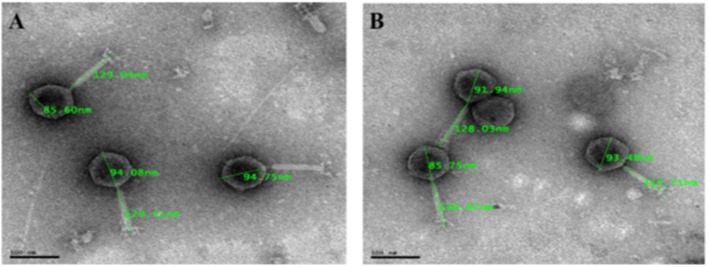
Transmission electron micrograph images of representative phage isolates negatively stained with 1% ammonium molybdate. Both phages (**A**: EC11B2PV; **B**: EC118BPV) belong to the myoviridae family and are showing icosahedral capsid and long contractile tail with tail fibers. The bars indicate scale (100 nm).

Eight selected phage isolates were subjected to TEM analysis to determine their morphotype. Transmission electron micrograph images of the phages and structural dimensions are shown in Figure 3 and Table 3, respectively. Phage isolates were classified as per the International Committee on Taxonomy of Virus (ICTV) classification based on the three-dimensional structure observed. All phage isolates showed similar morphotype on TEM analysis. Structurally, the phages had an icosahedral head and a neck attached to a long contractile tail, with tail fibers, and they were classified under the order Caudovirales, *Myoviridae* family. The phage icosahedral heads ranged from 81.2 ± 6 to 95.6 ± 3 nm while the contractile tails ranged from 118.1 ± 0.3 to 135 ± 2 nm. Phage EC10C2PV had the longest icosahedral head with a diameter of 95.6 ± 3 nm and the longest contractile tail of 135 ± 2 nm with fibers. Phage EC10C3PV had the smallest icosahedral head with a diameter of 81.2 ± 6 nm and the shortest contractile tail of 118.1 ± 0.3 nm with fibers.

**Table 3:** Phage dimensions based on transmission electron microscopy (TEM) analysis.

**Phage ID**	**Phage morphotype**				**Order**
	**Head structure**	**Head dimensions (nm ± stdv)**	**Tail structure**	**Tail dimensions (nm ± stdv)**	
EC366VPV	Icosahedral capsid	86.7 ± 2	Contractile sheath	120.3 ± 9	Caudovirales
EC11B2PV	Icosahedral capsid	91.5 ± 3	Contractile sheath	129.3 ± 0.2	
EC10C2PV	Icosahedral capsid	95.6 ± 3	Contractile sheath	135 ± 2	
EC366APV	Icosahedral capsid	88.5 ± 3	Contractile sheath	129.8 ± 3	
EC12APV	Icosahedral capsid	87.8 ± 2	Contractile sheath	121.9 ± 6	
EC118BPV	Icosahedral capsid	90.4 ± 3	Contractile sheath	123.9 ± 6	
EC3A1PV	Icosahedral capsid	85.6 ± 1	Contractile sheath	119.3 ± 1	
EC10C3PV	Icosahedral capsid	81.2 ± 6	Contractile sheath	118.1 ± 0.3	

### Phage Stability and Viability Against Different Temperatures

**Figure 4:**
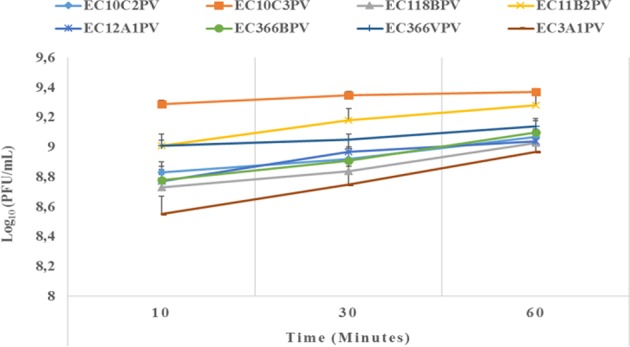
Effect of time on persistence (stability/survivability) of individual phages at 37°C. The error bars represent the standard deviation. PFU, plaque-forming unit.

**Figure 5:**
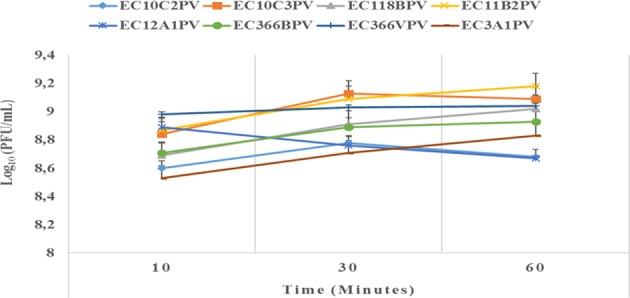
Effect of time on persistence (stability/survivability) of individual phages at 40°C. The error bars represent the standard deviation. PFU, plaque-forming unit.

The results showed a significant (*p* < 0.001) time × temperature interaction effect on the stability and viability of the phages. Incubation of phages from 10 to 60 min resulted in significant phage growth at 37°C (Figure 4). The growth from 10 to 30 min ranged from 8.55 to 8.75 log_10_ PFU/ml (at 37°C). Phage EC3A1PV revealed the lowest growth rate, while phage EC10C3PV exhibited the fastest growth rate from 10 to 60 min. Phage growth at 40°C when incubated for 10 to 60 min is depicted in Figure 5. From 10 to 30 min, seven phages revealed significant growth rates (ranging from 8.71 to 9.13 log_10_ PFU/ml). A decrease in phage EC12A1PV's growth rate was observed (from 10 to 60 min) while two phages (EC10C2PV and EC10C3PV) exhibited a decrease in growth rate from 30 to 60 min of incubation period.

**Figure 6:**
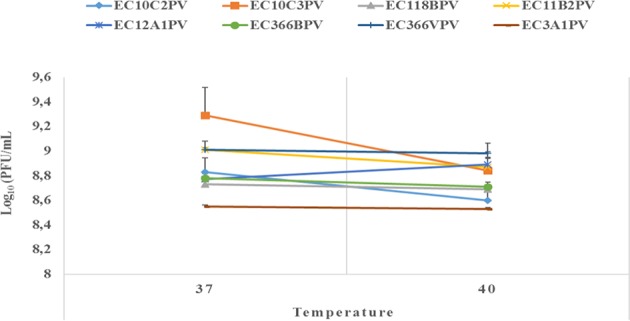
Survival and stability of individual phages when exposed to different temperatures for 10 min. The error bars represent the standard deviation. PFU, plaque-forming unit.

**Figure 7:**
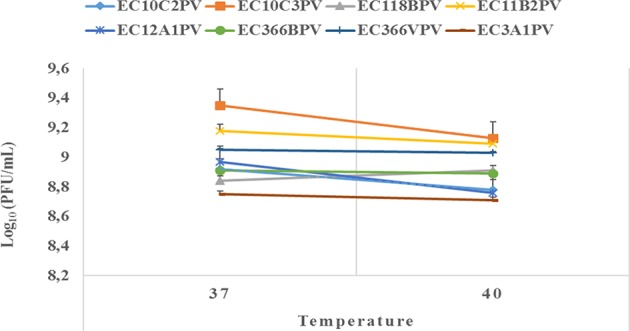
Survival and stability of individual phages when exposed to different temperatures for 30 min. The error bars represent the standard deviation. PFU, plaque-forming unit.

**Figure 8:**
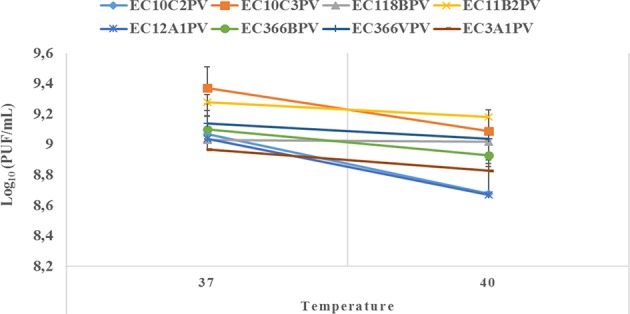
Survival and stability of individual phages when exposed to different temperatures for 60 min. The error bars represent the standard deviation. PFU, plaque-forming unit.

An exposure of individual phages to 37°C for 10–30 min showed a significant increase in growth rate with time (0.4–2.3% growth rate at 37°C; Figures 6, 7). The average growth rate of the phages increased from 0.2 to 0.17 log_10_ PFU/ml at 37°C (10–30 min of incubation period). Phages revealed various responses when incubated at 40°C. A decline in growth rate in other phages was observed. When incubated for 10 min at 40°C, EC10C3PV's growth rate declined the most by 4.8%, while phage EC3A1PV's growth rate was the least affected, showing only a 0.2% decline. After 30 min of incubation, a decline in growth rate was observed in phages EC366BPV (0.2%), EC366VPV (0.2%), and EC10C3PV (2.4%) (Figure 7). When incubated for 60 min at 40°C, phages EC10C2PV (0.28 log_10_ PFU/ml), EC10C3PV (0.39 log_10_ PFU/ml), and EC12A1PV (0.37 log_10_ PFU/ml) exhibited the greatest decline in growth rates when compared to their respective rates for 60 min at 37°C (Figure 8).

### Phage Stability and Viability Against Different pH Levels

**Figure 9:**
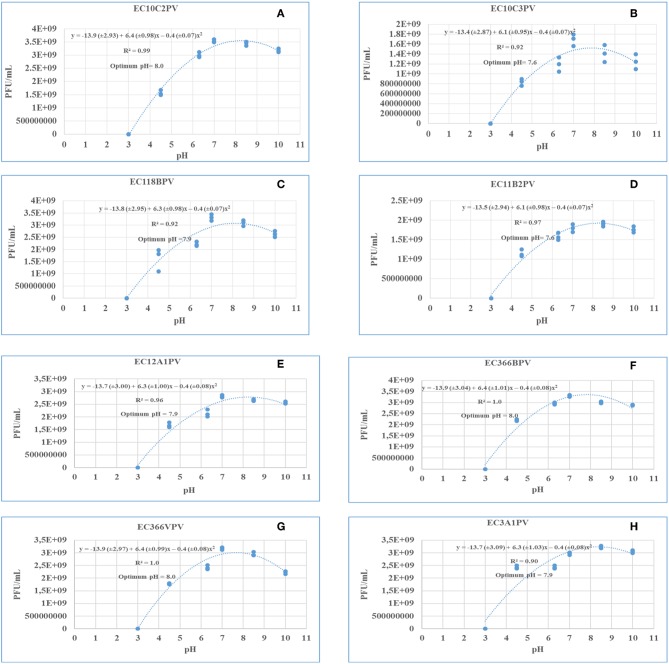
**(A–H)** Relationship between pH (*x*) and stability of phages [log_10_ plaque-forming unit (PFU), *y*] when incubated at 37°C for 24 h.

**Figure 10:**
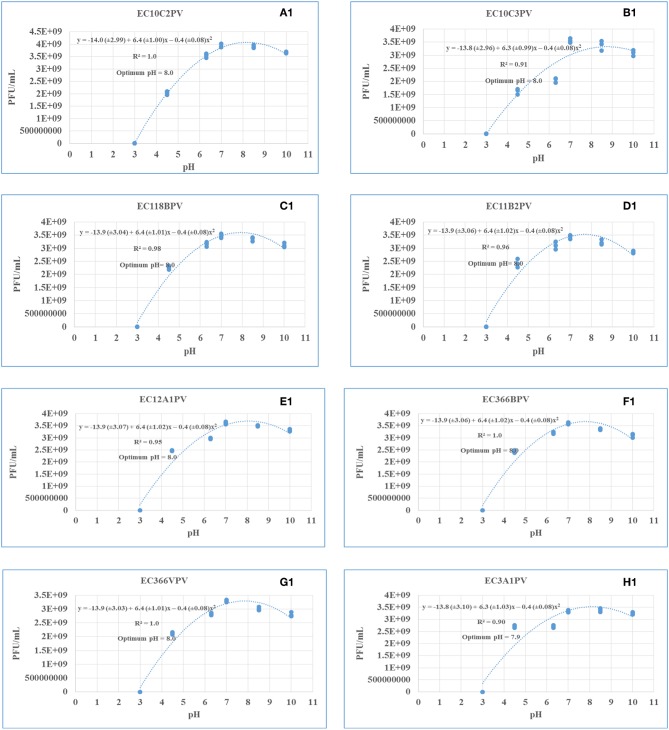
**(A1–H1)** Relationship between pH (*x*) and stability of phages [log_10_ plaque-forming unit (PFU), *y*] when incubated at 37°C for 48 h.

Response surface regression analysis revealed quadratic effects (*p* < 0.0001) of pH on phage stability when incubated for 24 and 48 h (Figures 9A–H, 10A1–H1, respectively). The pH optima for all the phages ranged from 7.6 to 8.0 with the *R*^2^ values ranging from 0.90 to 1.0 when incubated for 24 h (Figures 9A–H). Three phages showed maximum stability at pH 8.0 determined from the following quadratic equations: *y* = −13.9 (±2.93) + 6.4 (±0.98)*x* – 0.4 (±0.07)*x*^2^ (EC10C2PV), *y* = −13.9 (±3.04) + 6.4 (±1.01)*x* – 0.4 (±0.08)*x*^2^ (EC366BPV), and *y* = −13.9 (±2.97) + 6.4 (±0.99)*x* – 0.4 (±0.08)*x*^2^ (EC366VPV). Phages EC10C3PV and EC11B2PV exhibited maximum stability at pH 7.6, which was determined from the following quadratic equations: *y* = −13.4 (±2.87) + 6.1 (±0.95)*x* – 0.4 (±0.07)*x*^2^ and *y* = −13.5 (±2.94) + 6.1 (±0.98)*x* – 0.4 (±0.07)*x*^2^, respectively. When incubated for 48 h, pH optima for phage stability ranged from 7.9 to 8.0 with *R*^2^ values ranging from 0.90 to 1.0 (Figures 10A1–H1). Seven phages exhibited maximum stability at a higher (8.0) optimum pH while only one phage showed maximum stability at a lower (7.9) pH determined from the quadratic equation *y* = −13.8 (±3.10) + 6.3 (±1.03)*x* – 0.4 (±0.08)*x*^2^.

### One-Step Growth Curve Bacteriophages

**Figure 11:**
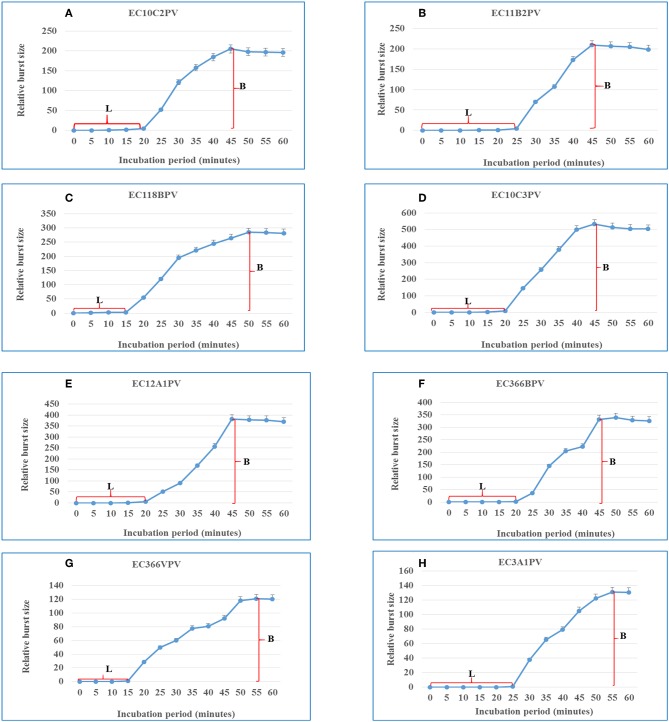
**(A–H)** One-step growth curves for eight *Escherichia coli* O177-specific phage isolates. Latent period (L) and burst size (B). The error bars indicate standard deviation.

A one-step growth curve analysis for the eight phage isolates was performed to determine the latent period and relative burst size per infected bacterial cell. Data generated were analyzed and used to construct triphasic curves (Figures 5–11A–H). The latent period for all the phages ranged from 15 to 25 min (average = 20 ± 3.8 min). Phages EC11B2PV and EC3A1PV had the longest latent period (25 min), while phages EC118BPV and EC366VPV had the shortest (15 min) latent period. The latent period for the other four phages was 20 min. In terms of burst size, phages EC10C3PV and EC12A1PV had the largest burst size per infected cell (522 and 367 PFUs, respectively), while phage EC366VPV had the smallest burst size (91 PFUs) per infected cell.

## Discussion

The emergence of antibiotic resistance in foodborne pathogens has revitalized interest in the possible exploitation of lytic bacteriophages as an alternative biocontrol strategy. Because of their ability to lyse multidrug-resistant pathogens, lytic bacteriophages are considered as a natural and green technology for food preservation and safety (29). The isolation, identification, and full characterization of the bacterial host is a prerequisite for the successful isolation of suitable lytic bacteriophages intended for biocontrol of antimicrobial foodborne pathogens (30). Furthermore, reliable, reproducible, and efficient methods need to be employed for selection of suitable phage candidates for biocontrol application (31). In this study, 31 lytic *E. coli* O177-specific bacteriophage isolates were successfully isolated from cattle feces using multidrug-resistant atypical enteropathogenic *E. coli* O177 as a host. Since cattle are the main reservoirs of the atypical enteropathogenic *E. coli* O177 strain, this supports the idea that phages are present in every ecosystem where their hosts exist (4). The phages exhibited clear and discrete plaques with different sizes. The plaque size ranged between small and large (1–2 mm, respectively) sizes while phage titers ranged from 6.2 × 10^5^ to 3.1 × 10^13^ PFU/ml. Interestingly, a large proportion (71%) of phage isolates produced large and clear plaques on their preferred hosts. These characteristics were similar to those reported for *E. coli* O157-, *Listeria*-, *Pseudomonas*-, *Salmonella*-, and *Vibrio*-specific phages (23, 32, 33). From a biocontrol point of view, strictly lytic phages with high titers are considered as ideal candidates for biocontrol application (4, 34).

Host range is one of the most important criteria when selecting phages intended for biocontrol of antimicrobial foodborne pathogens (35). Eight phages were selected to determine phage host range. The selection criteria were based on the lytic profiles, plaque clarity, and size of the phages. A spot test revealed that the phages were capable of infecting different *E. coli* strains from two different categories [environmental atypical enteropathogenic *E. coli* O177 and Shiga toxin-producing *E. coli* (*E. coli* O26 and *E. coli* O157)]. Clear plaques were predominantly observed on *E. coli* O177 and *E. coli* O26 serotypes. Interestingly, three phages exhibited clear plaques on *E. coli* O177, *E. coli* O26, and *E. coli* O157 strains, suggesting that these phages were polyvalent, infecting strains from two different categories. Despite this, no phage could infect all the ATCC strains and environmental *Salmonella* species tested in this study. This could be attributed to the fact that ATCC strains and *Salmonella* species lack specific receptors for phage attachment. Based on EOP analysis, phages revealed high efficiency (EOP ≥ 0.5) on the *E. coli* O177 strain. Despite the fact that all the phages revealed clear plaques on *E. coli* O26 and O157 strains on the spot test, only three phages exhibited medium to low EOP (<0.5) on *E. coli* O26 and O157 serotypes. This suggested that phages were highly specific to the *E. coli* O177 strain. Moreover, host specificity is regarded as a desirable characteristic for potent phage application, particularly in live animals to ensure that they have little or no impact on the beneficial gut microflora (2, 6). Furthermore, infectivity variation might be due to the non-specific binding receptors on the host cell wall or the presence of phage-resistant strains (6).

A negative staining procedure was used for TEM analysis. Based on TEM results, all eight phage isolates revealed a similar morphotype. Structurally, the phages had an icosahedral head and a neck attached to a long contractile tail with tail fibers connected to the baseplate. The icosahedral head of the phages ranged from 81.2 ± 6 to 95.6 ± 3 nm in size while the contractile tails ranged from 118.1 ± 0.3 to 135 ± 2 nm. Based on these characteristics, phage isolates were classified under the order Caudovirales and *Myoviridae* family (27). Moreover, these characteristics were similar to those of T1-7-like *E. coli* phages (4, 36). Given the fact that the *Myoviridae* family contains double-stranded DNA phages (4), all eight phages were presumptively classified under linear double-stranded DNA phages. The tail fibers contain proteins, which help the phage to recognize their specific receptors on the bacterial cell wall and thus restrict the phage from binding to non-specific bacterial cell (37). This explains the host specificity of the phages isolated in this study.

External factors such as pH and temperature may influence the stability and infectivity of the phages (7). These factors may fluctuate, particularly in live animals because of diet and/or ambient temperature. In view of this, phages intended for biocontrol application, particularly in live ruminants, must be tested against an appropriate range of pH and temperature. For this reason, the effect of exposure to different temperatures (37 and 40°C) for different times on infectivity and stability of eight phages was evaluated. Given that complete bacterial lysis by phage takes 20–40 min (33), phage growth at different temperatures was monitored after 10, 30, and 60 min. Furthermore, the incubation temperatures were selected because the temperature in the digestive system of the ruminant ranges from 37 to 40°C. The ability of phages to survive at these temperatures suggests that they can be applied in live animals as biocontrol agents. When incubated at 37°C, phages revealed a significant growth rate at each time point. Phages EC10C3PV, EC11B2PV, and EC366VPV revealed the fastest growth rates; phage EC3A1PV showed the slowest growth rate from 10 to 60 min. These results are similar to those reported in previous studies (28).

Phages revealed variable growth patterns when exposed to 40°C. Generally, phages showed a decline in growth rate at 40°C when compared to their growth rate at 37°C. When exposed to 40°C for 10–30 min, seven phages exhibited a significant growth rate. However, one phage revealed a decline in growth rate after 30 min while two phages, EC10C2PV and EC10C3PV, only exhibited a decline in growth from 30 to 60 min at 40°C. This demonstrates that these three phages were less stable at high temperature and, therefore, their application in live animals is limited because the rumen temperature is 39°C. Despite this, the other five phages were fairly stable at 40°C, suggesting they may be suitable for biocontrol application in live animals.

The response surface regression analysis revealed a significant relationship between pH and phage stability. The optimal pH for phages at different incubation times ranged from pH 7.6 to 8.0 (24 h) and pH 7.9 to 8.0 (48 h). When incubated for 24 and 48 h, all phages exhibited similar growth trends and survival over a wide range of pH (4.5–10.0). Despite this, all the phages were sensitive to low pH (3.0) with no activity being observed after 24 and 48 h of incubation at this pH. This is consistent with previous studies, which reported that exposure of phages to pH 3.0 and below significantly reduced the viability and stability of phages (38, 39). Although the optimum pH for all the phages is 7.6–8.0, phages revealed good stability even at lower pHs of 6.3 and 7.0, which encompasses rumen pH values (6.5–6.9). And this indicates their potential suitability for use in preharvest intervention strategies that may be designed for application in ruminants.

Phage latent period and burst size are important parameters to consider when selecting phages for biocontrol purposes (5, 31). Phages with a short latent period and large burst size are more effective in inactivating bacteria and are thus considered to be suitable for biocontrol application (35). One-step growth curves revealed that the eight phages have different patterns of growth, suggesting that they have distinct genotypes. They displayed outstanding characteristics such as short latent periods and large bust sizes, which make them attractive for the control of the *E. coli* O177 strain. The latent period of phages ranged from 15 to 25 min, while the burst size ranged from 91 to 522 PFU per host cell. In addition, the average latent period for all the phages was 20 ± 3.8 min while the burst size was 260 ± 144 PFU per host cell. These results were consistent with those reported previously (35). Two phages, EC118BPV and EC366VPV, had the shortest latent period (15 min) while EC11B2PV and EC3A1PV had the longest latent period (25 min). Phages EC10C3PV, EC118BPV, and EC12A1PV had the largest burst size per infected cell (522 and 367 PFU per host cell, respectively). Interestingly, these three phages also showed broad host range in the spot test. This demonstrates that these phages are better suited for biocontrol application (40).

In conclusion, lytic bacteriophages infecting the *E. coli* O177 environmental strain were successfully isolated in this study. Furthermore, phages were capable of infecting three *E. coli* strains from two different categories, atypical enteropathogenic *E. coli* (*E. coli* O177) and Shiga toxin-producing *E. coli* (*E. coli* O26 and *E. coli* O157). Despite this, no phage could infect ATCC strains and environmental *Salmonella* species tested. Considering strong lytic activity, broad spectrum, and stability at different temperatures and pH levels, phages isolated in this study are considered as potential candidates for *in vivo* control of the *E. coli* O177 strain. However, further studies using appropriate *in vitro* and *in vivo* models are required to evaluate the efficacy of *E. coli* O177-specific phages in reducing *E. coli* O177 in live animals and meat products. Moreover, whole-genome sequence analysis is also required to determine the presence of undesirable genes in these phages.

## Data Availability Statement

The datasets generated for this study are available on request to the corresponding author.

## Ethics Statement

The statement, Ethical clearance was obtained from the Faculty of Natural and Agricultural Sciences Ethics committee, North West University prior to the commencement of the study. An ethics number NWU-01223-19-S9 was assigned to the study.

## Author Contributions

VM and CA conceived and designed the experiments, contributed reagents, material, and analysis tools. PM performed the experiments. PM, VM, and CA wrote the paper and data analysis.

### Conflict of Interest

The authors declare that the research was conducted in the absence of any commercial or financial relationships that could be construed as a potential conflict of interest.
